# Can digital health researchers make a difference during the pandemic? Results of the single-arm, chatbot-led Elena+: Care for COVID-19 interventional study

**DOI:** 10.3389/fpubh.2023.1185702

**Published:** 2023-08-25

**Authors:** Joseph Ollier, Pavani Suryapalli, Elgar Fleisch, Florian von Wangenheim, Jacqueline Louise Mair, Alicia Salamanca-Sanabria, Tobias Kowatsch

**Affiliations:** ^1^Mobiliar Lab for Analytics, Chair of Technology Marketing, Department of Management, Technology, and Economics, ETH Zurich, Zurich, Switzerland; ^2^Centre for Digital Health Interventions, Institute of Technology Management, University of St. Gallen, St. Gallen, Switzerland; ^3^Centre for Digital Health Interventions, Chair of Information Management, Department of Management, Technology, and Economics, ETH Zurich, Zurich, Switzerland; ^4^Future Health Technologies, Singapore-ETH Centre, Campus for Research Excellence and Technological Enterprise (CREATE), Singapore, Singapore; ^5^Saw Swee Hock School of Public Health, National University of Singapore, Singapore, Singapore; ^6^Singapore Institute for Clinical Sciences (SICS), Agency for Science, Technology and Research (A^*^STAR), Singapore, Singapore; ^7^Institute for Implementation Science in Health Care, University of Zurich, Zurich, Switzerland; ^8^School of Medicine, University of St. Gallen, St. Gallen, Switzerland

**Keywords:** chatbot, conversational agent, COVID-19, holistic lifestyle intervention, pandemic lifestyle care, mental health, anxiety, depression

## Abstract

**Background:**

The current paper details findings from Elena+: Care for COVID-19, an app developed to tackle the collateral damage of lockdowns and social distancing, by offering pandemic lifestyle coaching across seven health areas: anxiety, loneliness, mental resources, sleep, diet and nutrition, physical activity, and COVID-19 information.

**Methods:**

The Elena+ app functions as a single-arm interventional study, with participants recruited predominantly via social media. We used paired samples *T*-tests and within subjects ANOVA to examine changes in health outcome assessments and user experience evaluations over time. To investigate the mediating role of behavioral activation (i.e., users setting behavioral intentions and reporting actual behaviors) we use mixed-effect regression models. Free-text entries were analyzed qualitatively.

**Results:**

Results show strong demand for publicly available lifestyle coaching during the pandemic, with total downloads (*N* = 7′135) and 55.8% of downloaders opening the app (*n* = 3,928) with 9.8% completing at least one subtopic (*n* = 698). Greatest areas of health vulnerability as assessed with screening measures were physical activity with 62% (*n* = 1,000) and anxiety with 46.5% (*n* = 760). The app was effective in the treatment of mental health; with a significant decrease in depression between first (14 days), second (28 days), and third (42 days) assessments: F_2,38_ = 7.01, *p* = 0.003, with a large effect size (η2G = 0.14), and anxiety between first and second assessments: t_54_ = 3.7, *p* = <0.001 with a medium effect size (Cohen *d* = 0.499). Those that followed the coaching program increased in net promoter score between the first and second assessment: t_36_ = 2.08, *p* = 0.045 with a small to medium effect size (Cohen *d* = 0.342). Mediation analyses showed that while increasing number of subtopics completed increased behavioral activation (i.e., match between behavioral intentions and self-reported actual behaviors), behavioral activation did not mediate the relationship to improvements in health outcome assessments.

**Conclusions:**

Findings show that: (i) there is public demand for chatbot led digital coaching, (ii) such tools can be effective in delivering treatment success, and (iii) they are highly valued by their long-term user base. As the current intervention was developed at rapid speed to meet the emergency pandemic context, the future looks bright for other public health focused chatbot-led digital health interventions.

## Introduction

Chatbots in digital health have become widespread in their application: from health services [e.g., symptom checking apps (1)] to interventions treating common mental disorders (CMDs) [e.g., anxiety (2), depression (3), burnout (4)], to those addressing non-communicable diseases (NCDs) [e.g., diabetes (5), obesity (6, 7), asthma (8)], amongst many others [see reviews: (9–13)]. Their ability to relay complex information in a communicative, dyadic manner whilst also facilitating relationship building efforts (14) [i.e., the working alliance (15)] has been noted as particularly conducive to health outcome success (8). When delivered via a smartphone app, chatbots can integrate useful tools and features to further coaching efforts (16), for example, the collection of sensor data (e.g., step counts) and tailored suggestions (e.g., “only 1,000 more steps needed to complete your goal!”) (17, 18). Like other digital services, chatbots are always available: fully adaptable to the schedule and circumstances of their focal user and relatively free from geographic, temporal, or other constraints (19). Chatbots therefore represent a low-cost, scalable tool that can relay treatment plans developed by clinicians and/or healthcare researchers (20–22), and within the pandemic context, may be particularly useful in addressing worsened health outcomes caused by stay-at-home orders (23, 24), especially for vulnerable subpopulations where lower health literacy, self-efficacy and/or access to health promoting resources exists (25, 26).

While chatbots have exhibited significant success across multiple treatment domains (11), their application as a tool to promote population-level health remains unexplored however (23). This is despite research showing that interventions targeting multiple facets of individuals' lifestyle are often highly effective (27). The diabetes prevention program (DPP), for example, uses diet and physical activity to control HBA1c and body weight, and social support groups to bolster mental health (28). While some chatbot-led interventions similarly target various lifestyle health areas [e.g., physical activity and cognitive behavioral therapy for suicide prevention (29) or diet and physical activity for gestational diabetes (30)], these interventions remain applied to a single treatment domain (i.e., to tackle a specific NCD/CMD) rather than tackling multiple health domains simultaneously (i.e., a lifestyle intervention). Thus, a major remaining challenge in digital health is the implementation of chatbot-led holistic lifestyle interventions (23): assessing and targeting areas of health vulnerability from individuals across the population, coaching them to address their current health needs (e.g., reducing anxiety), and encouraging the take-up of other health promoting behaviors (e.g., increasing physical activity). By doing so, such interventions have the potential to improve population level health and wellbeing, reducing pressure on strained healthcare systems (31, 32).

The current paper overviews findings from one such holistic lifestyle intervention, developed specifically for the COVID-19 pandemic context: Elena+: Care for COVID-19 (23). Elena+ addresses the collateral damage caused by the pandemic on public health [for example, requirements to stay at home and reduced physical activity (33), or alarming news stories and increased anxiety (34, 35)] via a chatbot-led psychoeducational coaching program. To do this, the Elena+ app assesses individuals' vulnerability across seven lifestyle health areas (anxiety, mental resources, loneliness, sleep, diet and nutrition, physical activity, and COVID-19 information) and recommends content from a lifestyle health coaching program, consisting of 43 subtopics completed over the course of approximately half a year. Since August 2020, Elena+ has been available free of charge on iOS and Android mobile devices in the United Kingdom, Ireland, Switzerland, and Android only in Spain, Mexico, Colombia, and the United States. As of 20th June 2022, when the final data download occurred, the intervention had 7,135 downloads.

In addition to its primary function as a publicly available coaching tool, the Elena+ project was also set up to contribute to the following research questions: First, to understand the health status of individuals downloading a pandemic-focused coaching app, as assessed through a gamified quiz upon first use of the app. Second, to track users changes in outcome assessments over time, for example, whether scores for a given topic (e.g., anxiety) decrease as individuals continue the coaching process. Third, as completing fewer pleasant activities has been linked with lower wellbeing during the pandemic (36), we examine whether behavioral activation, defined as patients increasing the “number of pleasant activities engaged in” (37), mediates the relationship between number of coaching sessions (i.e., subtopics) completed and outcome assessments for a given topic. To do this we investigate the match between behavioral intentions set at the end of a coaching session and actual behaviors reported one or more weeks later. Fourth, to assess user evaluations of the app with a combination of quantitative and qualitative measures, for example, changes in net promoter score (38) over time or free-text entries submitted by participants.

The Elena+ app was created to help address pressures on public health during the pandemic, and the following paper reports areas of success and failures: important lessons learned which can help developers of digital interventions should future public health emergencies arise. Additionally, by looking forward and focusing on holistic lifestyle health coaching, we begin a process of thinking differently about public health campaigns: as the adage goes, prevention is better than cure, and the additional benefit of holistic lifestyle coaching interventions such as Elena+ is that they may prevent NCDs and CMDs arising altogether. Lifestyle interventions can therefore begin the shift of a healthcare system designed to treat acute diseases of the 20th centuries (39) to the one which can pre-empt and prevent NCDs/CMDs diseases arising in a cost-effective manner (40). Thus, while Elena+ was developed for the pandemic context to coach individuals and encourage healthy behaviors, lessons learnt are highly transferable to other challenges of the 21^st^ century digital healthcare delivery.

## Methods

### Intervention design

Elena+: Care for COVID-19 uses a pre-scripted chatbot to guide users through a psychoeducational coaching program. A full overview of the intervention design is given in the Study Protocol paper by Ollier et al. (23), therefore, a briefer overview is offered in the current paper with the interested reader encouraged to refer to the full paper.

#### User journey

##### First use

Following download of the app, the journey of a new user is as follows: (i) individuals enter basic information (e.g., their nickname, age, gender), receive onboarding regarding the purpose of the Elena+ app, and give informed consent, (ii) users are encouraged to take a gamified quiz that acts as a tailoring assessment of their health status across the seven health areas, (iii) users are then directed to coaching materials matching their state of vulnerability, for example, if scoring 3 or more for the patient health questionnaire screening measure (PHQ-2) participants would be recommended loneliness and mental resources topics, (iv) users then progress to topic selection, complete a short one-time onboarding session for the chosen topic, and start a coaching session (i.e., subtopic), (v) at the end of the coaching session, individuals are asked to select a “tip” (i.e., behavioral intention) that they intend to apply in their real life, (vi) lastly, users set a date for their next coaching session (with the option to come back before the scheduled appointment by using the “wake up” button).

##### Continuing use

Upon next use of the app users begin at the: (i) “welcome back” dialogue, answering any survey questions as necessary, (ii) they then proceed to topic selection and complete a subtopic, (iii) and may then choose to select another subtopic or finish the session, (iv) after finishing, they set an appointment for a future session or may come back at any time using the “wake up button”. When participants have completed all subtopics within Elena+, a dialogue is triggered congratulating them and thanking them for their participation. After this point, individuals can continue to use the app and answer ongoing survey questions.

#### Intervention components

**Figure 1:**
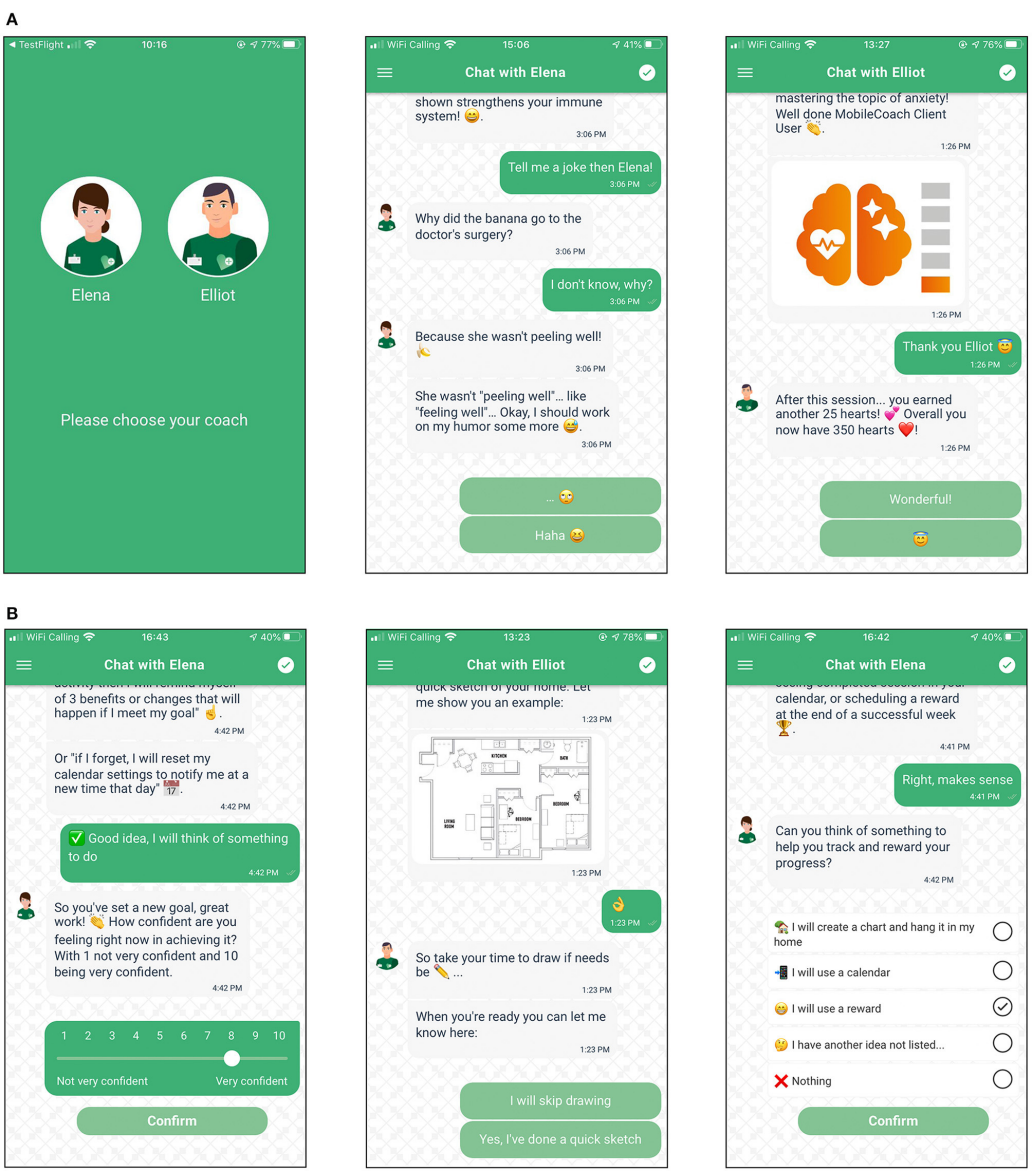
**(A)** Example screenshots of engagement intervention components. **(B)** Example screenshots of lifestyle intervention components.

The intervention consists of both engagement and lifestyle intervention components fully delivered by the chatbot without human support: the former were integrated to promote continued usage of the app and therefore adherence to the coaching program, whilst the latter aimed to boost participant health-literacy levels and encourage the formation/continuation of health promoting behaviors. Engagement intervention components included: (i) the interpersonal style of the chatbot [i.e., friendly, non-forceful and adhering to principles of positive psychology coaching and motivational interviewing (41–44) to enable the “working alliance” (45–48)], (ii) personalization [i.e., giving user choice where possible, for example, selecting a female (Elena) or male (Elliot) coach or skipping activities/questions (47, 49–51)], (iii) gamification [i.e., receiving badges and hearts for completing activities and continuing to use the app (52, 53)], (iv) framing of usage experience expectations [i.e., explaining rationale for activities and data protection measures (54)], and, (v) social media promotion [i.e., using social media to promote the app and recruit participants (55, 56)]. Lifestyle intervention components included: (i) psychoeducation [i.e., promoting health literacy via coaching materials created by domain experts (57)], (ii) behavior change activities [activities from coaching fields such as motivational interviewing, cognitive behavioral therapy (58, 59)], (iii) planning activities [behavioral supports to aid in goal formation (60)]. Screenshots of example engagement intervention components and lifestyle intervention components can be viewed in Figures 1A, B.

#### Coaching content

Coaching content for each topic was created by a multi-disciplinary team of researchers and domain experts in seven different health areas. Each topic (e.g., Sleep) consisted of a variety of sub-topics (e.g., “What is sleep hygiene?”), typically requiring ~5–10 min to complete. Following the health action process approach (HAPA) (61), COVID-19 information, sleep, diet and nutrition, and physical activity were divided into beginner and intermediate+ difficulty levels, so that users could focus on coaching materials in line with their current knowledge and behavioral experience. For mental health topics, coaching sessions were not dichotomized into beginner and intermediate+ in order to better comply with evidence-based transdiagnostic treatments in mental health (62–64). Due to time constraints, diet and nutrition was only implemented with three coaching sessions. A full overview of the subtopics contained within each topic can be found in the Supplementary material or the study protocol paper (23).

###  Study design

The Elena+ app was set up as a single-arm interventional study, with ethical clearance given by the ETH Zurich university ethics board (application no: EK 2020-N-49) and reviews by Apple and Google. As the intervention has no control group (because participants download the app directly from the app stores), the authors describe the sample of users and how their progression through coaching content over time influences healthcare and user experience outcomes. The intervention timeframe was intended to last for approximately half a year, however, time to complete content was dependent on how fast users' chose to complete coaching content. All data were collected in app, and surveys were conducted by the chatbot directly. As Elena+ is a large and multi-faceted lifestyle care intervention, the study design can be broken down into the following sections:

#### Participant background and health status assessment

To detail the participant background, we collect basic socio-demographics on first use (i.e., after download) and again before fifth subtopic completion. We additionally assess screening measures from the gamified quiz (i.e., tailoring assessment) used to recommend coaching content to individuals based on their vulnerability scores in the different health areas. Individuals may also share free text regarding their reason for downloading the app.

#### Lifestyle care assessment

As the core research outcomes of the intervention, the study gathers information on participant health outcome assessments (OA) over time (for each health area where the participant begins a subtopic), the behavioral intentions (BI) set at the end of each coaching session (i.e., “tips”), and the self-reported actual behaviors (AB) (i.e., whether individuals report implementing those tips in their daily lives). The first OA follow-up occurred ~14 days after completion of a subtopic for a given health topic, with the 2^nd^ and 3^rd^ follow-ups occurring an additional 14 days thereafter, before the time interval was doubled to 28 days for subsequent OAs. In a similar manner, the first AB follow-up for a given subtopic occurred 7 days after setting BIs, with the 2^nd^ and 3^rd^ follow-ups measured 14 days after, before the time interval was again doubled to 28 for subsequent follow-ups. To reduce participant burden, we added some variation in the timing across topics to reduce the chance of multiple OA and AB questions from different topics co-occurring on the same day. An outline of the exact timing schedule of OA and AB questions can be found in the Supplementary material.

#### User experience assessment

To assess the user experience, we ask questions on a randomized basis after completion of a subtopic from the technology acceptance model (TAM) (65, 66), the session alliance inventory (SAI) (67), net promoter score (NPS) (38, 68), and a free-text entry option.

###  Recruitment

Elena+ is a publicly available tool available free of charge on iOS and Android mobile devices in the United Kingdom, Ireland, Switzerland, and Android only in Spain, Mexico, Colombia, and the United States. It is available in three language formats: English, European Spanish, and Latin American Spanish, with users only aged 18 or above allowed to use the app. The app can be found by searching for keywords on the iTunes and Android app stores such as COVID-19, coronavirus, mental health, sleep, exercise, diet, nutrition, coaching (and Spanish equivalents), or the full app name “Elena+: Care for COVID-19” (Spanish: “Elena+ cuidados ante la COVID-19”). This facilitates a degree of natural recruitment via individuals seeking health support apps, however, social media advertisements were also run-on Facebook periodically between April 2020 and December 2021 in the U.K., Ireland, U.S.A, Spain, Colombia, and Mexico. The campaigns resulted in total 4 994 downloads, with a total of 9 737 USD spent.

###  Measures

#### Tailoring assessment

The tailoring assessment followed verified short form screening measures from established literature wherever possible to reduce participant burden, particularly as the assessment occurred during first use of the app. Where short form screening measures did not exist, we asked team members with expertise in a given health area to suggest items to use, with further discussions amongst the wider team [consisting of 43 members, see study protocol paper (23)] used to corroborate their inclusion. For example, as no short form insomnia severity index (ISI) exists, we selected two items from the full seven-item ISI that the mental health team assessed as being most directly applicable to insomnia. Similarly, to assess COVID-19 related knowledge, we utilized resources from the World Health Organization website (69). Due to time constraints in delivering the app quickly to meet with the pandemic context, we were unable to further test these short form measures prior to use however. Vulnerability assessments were made based on cut-off criteria from established literature wherever available, when this was not possible, recommendations were again made based on the best judgement of the team responsible for a given health area. Table 1 outlines the tailoring assessment measures and scores classified as showing vulnerability which were used to recommend coaching content for users.

**Table 1:** Tailoring assessment constructs used to assess vulnerability.

**Topic**	**Construct**	**Vulnerability**	**No. of items**	**References**
Anxiety	General anxiety disorder (GAD)	3 or higher (0–6)	2	(118)
Loneliness	Patient health questionnaire (PHQ)	3 or higher (0–6)	2	(117)
Mental resources	Patient health questionnaire (PHQ)	3 or higher (0–6)	2	(117)
Sleep	Insomnia severity index (ISI)	5 or higher (0–8)	2	(130)
Physical activity	Knowledge and awareness of physical activity guidelines	3 or less (1–6)	2	(131)
	Stage of Change for Physical Activity Questionnaire Instrument	–	–	(132)
Diet and nutrition	Short consumer-oriented nutrition knowledge questionnaire	2 or less (0–4)	4	(133)
COVID-19 information	World Health Organization COVID-19 guidelines	1 or less (0–3)	3	(69)

#### Health outcome assessments

Health outcome assessments used established measures to assess changes in health outcomes over time, however, remained as short as possible to not over burden participants with the longest measures containing no more than seven items. All topics had a single instrument with multiple items (e.g., the General Anxiety Disorder 7-item for anxiety), with the exception of physical activity which contained two constructs: hours active (two items) and sedentary behavior (one item), as commonly applied in survey based physical activity assessments (70). The instruments used, no. of items per instrument, and relevant references are displayed in Table 2.

**Table 2:** Health outcome assessments.

**Topic**	**Instrument**	**No. of items**	**References**
Anxiety	General anxiety disorder: GAD-7	7	(118)
Loneliness	UCLA loneliness scale: ULS-6	6	(134)
Mental resources	Brief resilience coping scale	4	(135)
Depression	Patient health questionnaire: PHQ-2	2	(117)
Sleep	Insomnia severity index: ISI-7	7	(130)
Physical activity	Single-item physical activity measure/international physical activity questionnaire short form	3	(136–138)
Diet & nutrition	Short survey instruments for children's diet and physical activity: the evidence	4	(139)
COVID-19	COVID-19: risk perception and coping strategies	5	(140)

#### User experience assessments

We selected single-item versions of constructs for technology acceptance model as used in previous studies (65). NPS is single-item by design (38, 68). As the working alliance is a more complex construct, and no widely accepted single item exists, we utilized the 6-item session alliance inventory created by Falkenström et al. (67) developed for use as a repeated-measure post therapy session.

#### Behavioral assessments

Behavioral intention setting consists of choosing tips from multiple available options. An individual may select from a single tip, multiple, or no tips at all. Actual behaviors were measured by referring to the exact same answer options. An example screenshot of BI setting can be viewed in the Supplementary material. In the current intervention, BA was conceptualized as scheduling and completing activities (37) which are both pleasant and promote perceptions of mastery (71). To operationalize BA across subtopics, we summed the number of times a perfect match between BIs and ABs occurred within a topic: for example, the anxiety module consisted of five subtopics, thus a participant could have received a score from 0 to 5 for the anxiety BA. A perfect match was defined as reporting the exact same AB as the BI selected ~7 days or more prior. The rationale was that if individuals truly exhibited BA, they would clearly remember the BI they had set and report it exactly. An ordinal BA variable was thus created for each of the seven health topics. An additional BA variable was calculated using all subtopics that could range from 0 to 43. This resulted in a total of eight BA variables.

###  Statistical analysis

#### Health outcome assessments and user experience evaluations

To examine changes in OA and user experience assessments over time, we used either paired samples *T*-tests or within-subjects ANOVAs [with Geisser-Greenhouse correction applied where violations of sphericity existed (72)] depending on the number of available responses. A power analysis using G^*^Power version 3.1 (73) indicated that for a paired-samples *T*-test with two-tailed hypotheses to reach 80% power at a significance criterion of α = 0.05, a sample size of *n* = 26 would be required for a medium effect size (Cohen *d* = 0.5). For a within-subjects ANOVA with three measurements and a medium effect size (*F* = 0.3), a sample size of *n* = 20 was required to meet significance criterion of α = 0.05 at 80% power. Prior to running analyses, outliers were removed using the interquartile range method (74) and normality was verified using Shapiro-Wilk tests. Where sufficient sample size was attained for an OA, we first ran analyses using complete cases (i.e., individuals that have valid responses in all time periods examined), and then again under the intention-to-treat principle using the last observation carried forward (LOCF) method for participants who dropped out (75, 76). The European Medicine Agency states LOCF can be useful in providing a conservative estimate of the treatment effect, providing the patient's condition is expected to improve over time as a result of the intervention's treatment (77).

#### Mediating role of behavioral activation

To investigate the mediating role of BA between the number of coaching sessions completed and changes in OAs for a given topic, we also conducted a series of mediation analyses using repeated measures mixed-effect regression models, whereby outcomes over time were considered nested in the participant (78). The rationale for using a mixed-effects models was to better incorporate participant heterogeneity by specifying random intercepts and/or slopes per participant (78, 79). Rule-of-thumb sample size guidelines for two-level mixed-effect models require a minimum of 20 participants per time period (80), with 30 or more are desirable (81).

The first model (that investigated the mediator, BA) was a generalized linear mixed-effects model regressing BA for the topic on: (i) no. of subtopics completed, (ii) topic vulnerability (i.e., whether an individual had been assessed as vulnerable in this health area during the tailoring assessment), and (iii) assessment period. As BA is an ordinal dependent variable that counts the no. of times BA occurred, the model was specified using a Poisson distribution with a log link function (79). For interpretability, we display incidence rate ratios (IRR) for each regressor rather than raw coefficients in log form, whereby IRR values greater (less) than 1 indicated higher (lower) incidence of BA occurrence. The second model type (that investigated the dependent variable, OA) was a linear mixed effects model, regressing OA for a given topic on the same independent variables as the first regression model, however, with the addition of the mediating variable BA. As IRR in the first model are unstandardized by their nature, to aid comparability between model types we display unstandardized estimates for second model also.

For both regression model types, we ran four mixed-effect model types before selecting the preferred model. These were: (i) intercept only, (ii) random intercept per participant, (iii) random slope per participant, and (iv) random intercept and slope per participant. The fixed effects included were assessment period, topic vulnerability, and no. of coaching sessions completed. To select between models, we followed the approach in Peugh (78) and performed sequential chi-squared differencing tests on the four model types and additionally inspected Akaike information criterion (AIC), Bayesian information criterion (BIC), log-likelihood and deviance for model fit while favoring more parsimonious (i.e., less complex) models.

To assess whether mediation occurred, we used a Causal Mediation Approach (82), specifying no coaching sessions completed (i.e., subtopics) as the treatment variable, contrasting low treatment level (one completed coaching session) vs. high treatment level (maximum no. of completed coaching sessions in a given topic area). Average causal mediation effects (ACME), average direct effects (ADE) and total effects were calculated using bootstrapping with 1,000 re-samples.

#### Text analyses

Free-text entries from open questions at various points of the intervention were analyzed qualitatively. Based on the relevant literature, 2–3 raters (coders) are recommended as best practice (83, 84), with more than that not suggested (83). In this study, the code process was initially handled by one researcher (AS) and verified by another (JO). It is common for an experienced investigator to create the codes, and another to apply them. AS has been trained in qualitative research and has previous experience using the methodology. For ensuring objectivity, an open dialogue between researchers was a key to ensure the code reliability. Spanish comments were translated to English by AS.

The analysis involved the development of a content-oriented coding scheme classification (85), where recurrent words were grouped into categories. One author (AS) reviewed the open text from the users and corrected any spelling (but not grammar) errors before starting the coding process. The codes were short labels that represented important features of the data relevant to answering the research questions. The final list of codes generated by AS were reviewed by JO, who made suggestions on whether some codes could be merged or separated based on JOs independent classification of codes. AS finalized the decisions and grouped the final codes together into a set of overarching categories, before calculating the frequency of words per category and creating figures. To further elucidate the findings, we also extracted raw text comments (with spelling errors corrected) from users as quotations.

#### Deviations from planned analyses

It should be noted that the current paper deviates from planned analyses outlined in the study protocol by Ollier et al. (23) in two ways: First, auto-regressive moving average models (ARMA) were originally proposed for the analyses as they allow the possibility of creating forecasts based upon independent variable values and/or errors terms when splitting the dataset into training and test data sets (86). ARMA models however require larger sample sizes than were available from the data collected, with minimum 50 but preferably over 100 observations for each time point (87). Thus, we instead used *t*-test/ANOVA to establish changes in OA over time and used mixed-effect regression models to explore the mediating role of BI and AB, both of which confer to relevant sample size guidelines. Second, cluster analyses were planned to be used on marker variables [i.e., user dialogue choices with the chatbot, see study protocol paper (23)] collected during the intervention, however, sample size limitations meant that a smaller proportion of users set multiple values for the various marker variables across the interventions than anticipated. Thus, we instead explored marker variables descriptively. Analyses of the marker variables can be found in the Supplementary material.

## Results

###  Participant background

In total, 7,135 individuals downloaded the Elena+ app, with 55.8% opening the app (*n* = 3,928), and a subsequent 9.8% beginning the lifestyle intervention content by completing a subtopic (*n* = 698). Of these 698 users, the average number of subtopics completed was 3.1, and the average number of whole topics completed was 0.4.

**Figure 2:**
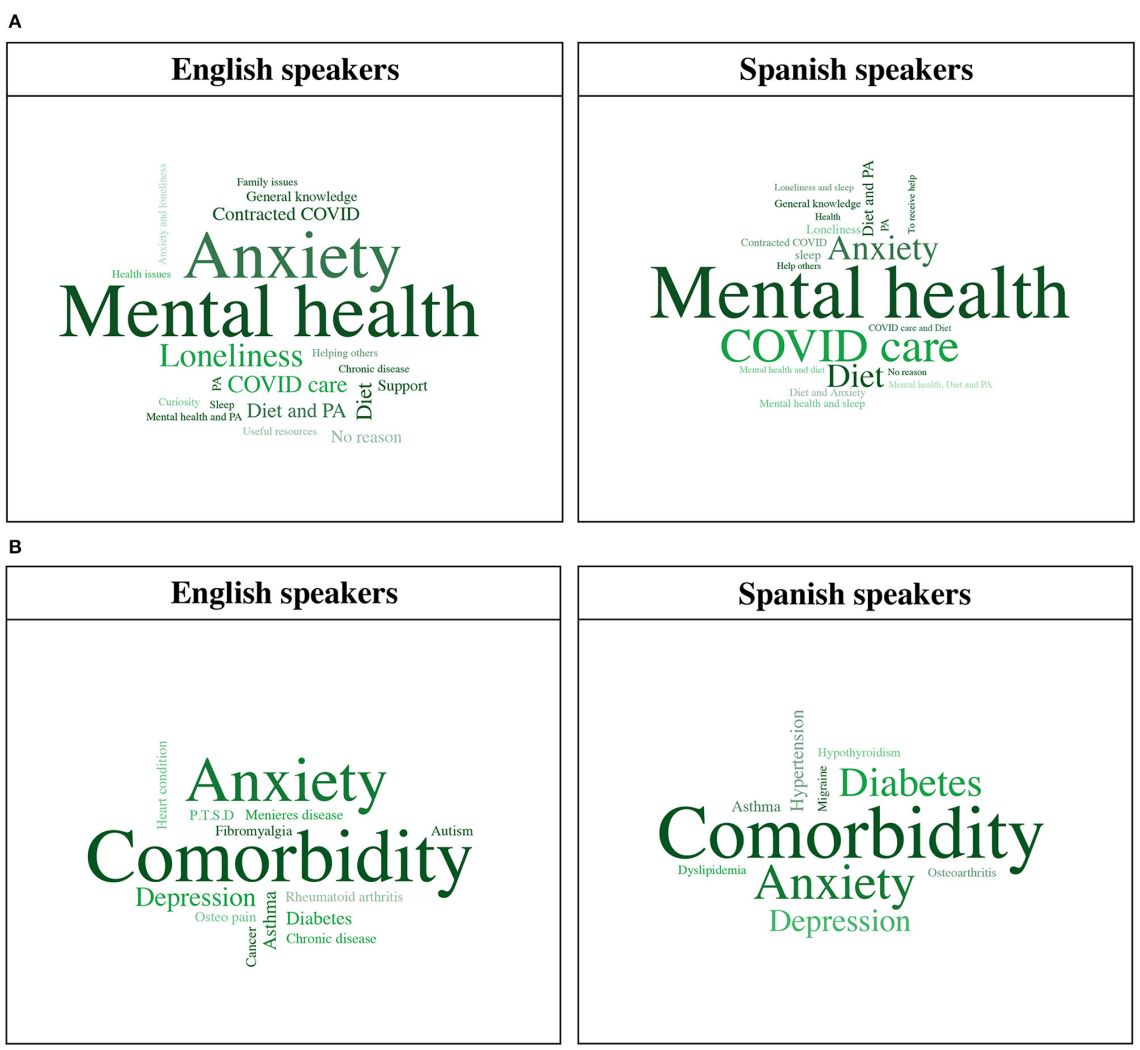
**(A)** Reasons for downloading Elena+ for English and Spanish speakers. **(B)** Chronic disease category classification for English and Spanish speakers. PA, physical activity; *P*.T.S.D, post-traumatic stress disorder.

Immediately after individuals began the conversation with the chatbot, we asked participants what topic they were most interested in. From a total of 1,614 responses, most popular topics, ordered by frequency were: anxiety (*n* = 692, 42.9%), physical activity (*n* = 239, 14.8%), diet and nutrition (*n* = 192, 11.9%), sleep (*n* = 176, 10.9%), mental resources (*n* = 142, 8.8%), COVID-19 information (*n* = 103, 6.4%), and loneliness (*n* = 70, 4.3%). When asked if they would like to share free text regarding their reason for downloading the app, 38% of Spanish (*n* = 205) and 29.2% English speakers (*n* = 81) wrote text related to “mental health” as their sole interest. When expanding mental health classification to also include related categories (e.g., loneliness, anxiety) and comorbidities (e.g., mental health and physical activity) 56.9% of Spanish speakers (*n* = 307) and 66.1% of English speakers (*n* = 181) wrote free text relating to mental health. Some quotations from the users include: “*Because I have really got Anxiety, Stress and Depression*.”, “*Interest in counselling and mental health*”, and “*I suffer from anxiety for some years now. Even though during the pandemic outbreak my anxiety was pretty well under control, I feel now how I struggle at nights with very intense fears*”. Reasons for downloading Elena+ for English and Spanish speakers are pictorially represented in Figure 2A. Full frequency counts for categories with example quotes are available in the Supplementary material.

Basic descriptive statistics collected during the initial onboarding conversation are displayed in Table 3. Summary statistics are organized by how far participants got into the intervention: (i) dropouts (0 subtopics completed), (ii) tentative users (1–4 subtopics completed), users (5–29 subtopics completed), and (iv) super users (30–43 subtopics completed). Findings showed that the user base was: (i) middle-aged, with a slight increase in mean age between dropouts and super users (ii) predominantly female, (ii) that more Spanish speakers downloaded the app, but the ratio of languages was relatively balanced, and (iv) that the content offered in Elena+ met the expectations of most users.

**Table 3:** Basic descriptive statistics.

**Intervention progress**		**Dropouts**	**Tentative users**	**Users**	**Super users**
Subtopics complete (*n*)		0	1–4	5–29	30–43
Participants (*n*, %)		923 (100)	583 (100)	99 (100)	9 (100)
Age (mean, SD)		43.8 (13.4)	44.3 (14.1)	47.4 (14.6)	49.6 (7.8)
Gender (*n*, %)	Male	329 (35.6)	197 (33.8)	19 (19.2)	2 (22.2)
	Female	583 (63.2)	382 (65.5)	78 (78.8)	7 (77.8)
	Other	11 (1.2)	4 (0.7)	2 (2)	0 (0)
Language (*n*, %)	English	294 (31.9)	229 (39.3)	44 (44.4)	5 (55.6)
	Spanish	629 (68.1)	354 (60.7)	55 (55.6)	4 (44.4)
Expectation met (*n*, %)	No	210 (22.8)	131 (22.5)	29 (29.3)	1 (11.1)
	Yes	713 (77.2)	452 (77.5)	70 (70.7)	8 (88.9)

The additional socio-demographics collected after the 5th subtopic completion had in total 240 responses. This showed that 35% of respondents (*n* = 84) had university level education, 27.5% were in full-time work (*n* = 66), and when asked about their health-status, participants rated their current health as adequate (mean 2.4, SD = 1.03). When asked if they were currently living with a chronic disease, 60.1% (*n* = 146) of individuals self-rated that they had at least one. When asked what their chronic disease was, top category classification was having a comorbidity (i.e., two or more conditions simultaneously) for both English (*n* = 15, 34.0%) and Spanish (*n* = 21, 35.0%) speakers, followed by anxiety with 27.3% (*n* = 12) and 21.7% (*n* = 13), respectively. Chronic disease free text entries amongst English and Spanish speakers are pictorially visualized in Figure 2B. Full frequency counts of chronic disease category classification are available in the Supplementary material.

###  Tailoring assessment

The number of individuals completing the tailoring assessment for different health areas varied due to dropouts or non-response. Table 4 displays the number of completed tailoring assessments per health topic, ordered by the number of individuals classified as vulnerable. Almost two thirds of individuals completing the physical activity assessment were classified as vulnerable (62%, *n* = 1,000) and almost half of individuals were classified as vulnerable in the anxiety assessment (46.5%, *n* = 760). It is also worth noting that 16.3% of individuals (*n* = 266) received the maximum score on the GAD-2 measure, and 6% (*n* = 97) for the PHQ-2 measure, and were recommended to immediately seek human support by the chatbot. For the other topics, approximately one third of individuals were classified as vulnerable, except for diet and nutrition (18.8%, *n* = 330) where numbers of vulnerable individuals were lowest from all topics. A figure displaying the full frequency of tailoring assessment scores can be found in the Supplementary material.

**Table 4:** Summary of tailoring assessment results.

**Topic**	***N***	**No. classified as vulnerable (*n*, %)**
Physical activity	1,614	1,000 (62)
Anxiety	1,636	760 (46.5)
Depression	1,624	548 (33.7)
COVID-19 information	1,907	635 (33.3)
Sleep	1,645	496 (30.2)
Diet and nutrition	1,753	330 (18.8)

###  Health outcome assessments

**Figure 3:**
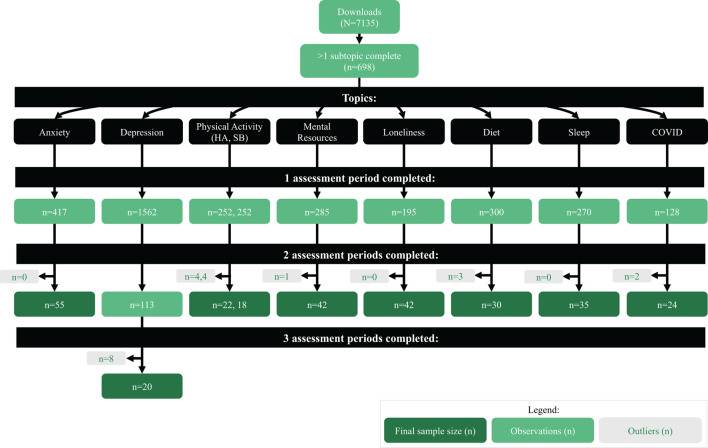
Flowchart of outcome assessment responses by assessment periods for each health topic. HA, hours active; SB, sedentary behavior.

Due to sample size limitations (i.e., sufficient numbers of individuals answering OAs over time), only two-tailed paired samples *T*-tests were possible for each of the health areas with the exception of PHQ-2 (depression symptoms) collected for all participants regardless of topic area (which had sufficient sample size for an ANOVA). Figure 3 outlines the number of valid outcome assessment responses for each time period, outliers removed prior to conducting the analyses and the final sample size used.

**Figure 4:**
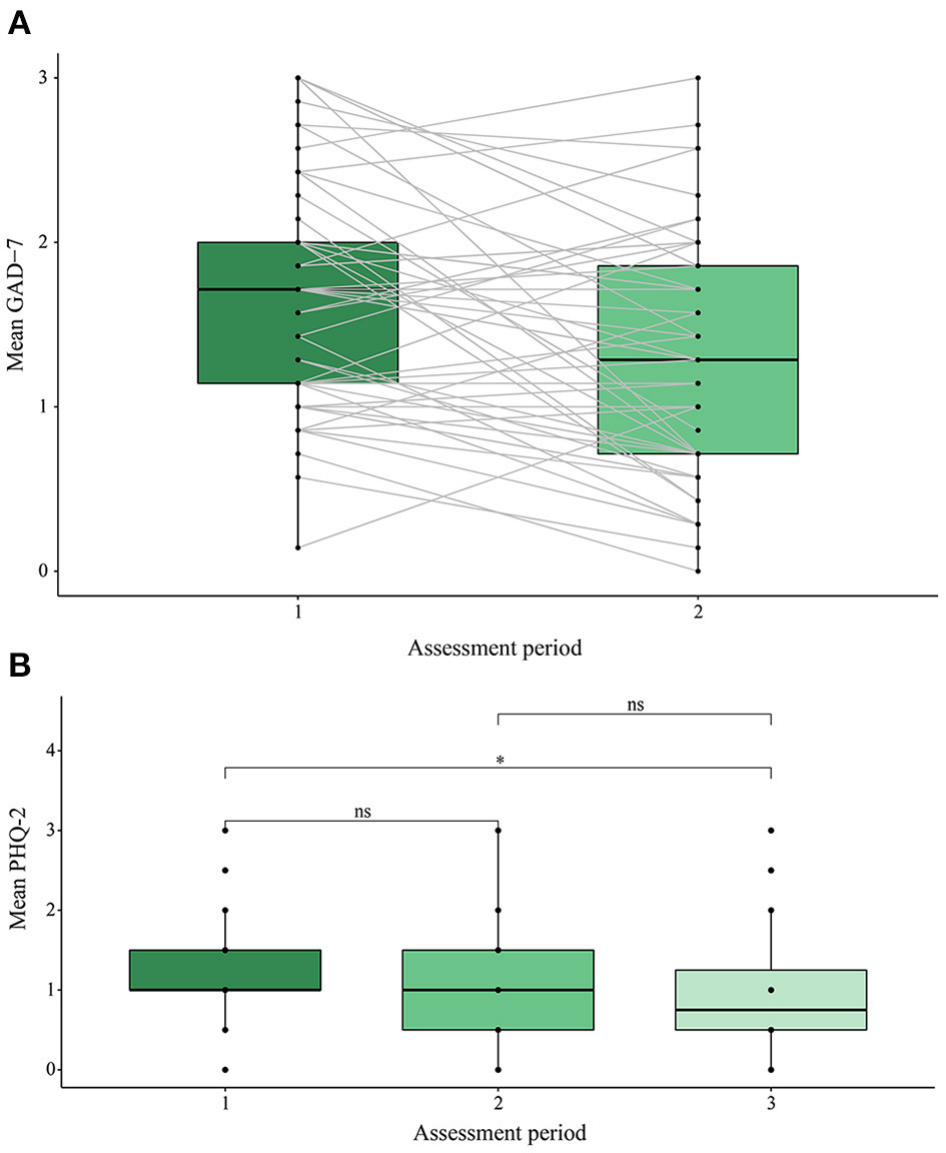
**(A)** Anxiety outcome assessment score in assessment periods 1 and 2. **(B)** Depression outcome assessment score across assessment periods 1, 2, and 3. GAD-7, general anxiety disorder 7-item instrument; PHQ-2, patient health questionnaire 2-item instrument. *p < 0.05, ns, non-significant.

Results of the complete cases analysis for each topic are summarized in Table 5A. The anxiety module showed a significant decrease in anxiety levels between assessment period 1 and 2, t_54_ = 3.7, *p* < 0.001, Cohen *d* = 0.499, with a medium effect size, as shown in Figure 4A. All other health topics exhibited no significant change between assessment period 1 and 2. It should also be noted that the topics COVID-19 information and physical activity failed to reach the sample size threshold based on the power analysis. Analysis of the anxiety module based on the intention-to-treat principle showed a significant decrease in anxiety between assessment period 1 and 2, t_416_ = 3.4, *p* < 0.001, Cohen *d* = 0.165, however, with a negligible effect size.

**Table 5:** Results of two-tailed paired samples *T*-tests for each health topic **(A)** and user experience construct **(B)**.

	**Period 1**	**Period 2**			
	**Mean (SD)**	**Mean (SD)**	***n***	***p***	**Cohen** ***d***
**(A) Health topic**					
Anxiety	1.66 (0.70)	1.31 (0.71)	55	< 0.001	0.499
Loneliness	2.80 (0.63)	2.79 (0.61)	42	0.831	0.033
Mental resources	3.3 (0.68)	3.24 (0.87)	42	0.921	0.015
Sleep	2 (0.78)	1.89 (1.02)	35	0.208	0.217
Diet and nutrition	3.09 (0.41)	3.11 (0.48)	30	0.837	0.038
COVID-19 information	3.69 (0.67)	3.82 (0.56)	24	0.341	0.199
Physical activity: hours active	1.07 (0.85)	0.91 (0.71)	22	0.431	0.171
Physical activity: hours sedentary	6.89 (2.61)	6.78 (2.46)	18	0.847	0.046
**(B) User experience construct**					
Perceived usefulness	6.27 (0.67)	6.48 (0.67)	33	0.069	0.326
Perceived ease of use	6.51 (0.69)	6.7 (0.52)	37	0.07	0.307
Perceived control	6.14 (0.8)	6.11 (1.17)	36	0.872	0.027
Perceived enjoyment	6.37 (0.6)	6.49 (0.61)	35	0.324	0.169
Net promoter score	8.51 (1.77)	9.08 (1.42)	37	0.045	0.342
Session alliance inventory	3.91 (0.93)	3.98 (1.01)	38	0.47	0.118

For the measure of depression, we conducted a within-subjects ANOVA (*n* = 20) to examine changes between assessment periods 1, 2, and 3. Results showed that a significant change in depression scores occurred: *F*_2,38_ = 7.01, *p* = 0.003, with a large effect size (η2G = 0.14). *Post-hoc* analyses with a Bonferroni adjustment revealed significant pairwise differences (*p* = 0.018) between assessment period 1 (mean 1.15, SD 0.43) and period 3 (mean 0.6, SD 0.598) and weakly significant differences (*p* = 0.094) between periods 1 and 2 (mean 1.02, SD 0.75). Results are displayed in Figure 4B. Analysis of depression using the intention-to-treat principle with a Greenhouse-Geisser correction applied [due to violations of sphericity, as recommended practice (72)] showed non-significant change in depression outcomes between assessment periods 1, 2 and 3, *F*_1.32, 2, 056.87_ = 0.166, *p* = 0.753, with a negligible effect size (η2G = < 0.001).

###  Mediating role of behavioral activation

#### Anxiety

Sequential chi-squared differencing tests and inspections of AIC, BIC, log Likelihood and deviance figures showed that the random intercept model exhibited best fit whilst being the most parsimonious solution for both the mediator model (BA): Δχ2(3) = 52.44, *p* < 0.001, and dependent variable model (OA): Δχ2(4) = 29.25, *p* < 0.001. A comparison of the fit indices for various models can be found in the Supplementary material. We therefore used estimates from these model specifications in the subsequent mediation analysis. Results from the random intercept anxiety BA and OA models are shown in Table 6A. Findings show that as the number of anxiety subtopics completed increased, the number of incidents of anxiety topic behavioral activation also increased (*p* < 0.001, IRR = 1.942) indicating that for every anxiety subtopic completed, behavioral activation for the anxiety topic was 1.942 times higher. Examining the anxiety outcome assessments showed that anxiety behavioral activation was an insignificant predictor of changes in anxiety outcome assessments (*p* = 0.702, *B* = −0.026), as was number of anxiety subtopics completed (*p* = 0.393, *B* = 0.056). However, anxiety vulnerability status was significant, responsible for higher anxiety scores (*p* < 0.001, *B* = 0.636). As confirmed in the previous section, users' anxiety reduced between assessment period 1 and 2 (*p* < 0.001, *B* = −0.351).

**Table 6:** Anxiety **(A)** and depression **(B)** model coefficients.

	**Behavioral activation (anxiety)**			**Outcome assessment (anxiety)**		
**Predictors**	***IRR***	***SE***	***p***	***B***	***SE***	***p***
**(A) Anxiety model**						
(Intercept)	0.115	0.050	< 0.001	1.498	0.231	< 0.001
Assessment period	1.000	0.164	1.000	−0.351	0.095	< 0.001
Subtopics completed (anxiety)	1.942	0.153	< 0.001	0.056	0.066	0.393
Vulnerability status (anxiety)	0.909	0.196	0.657	0.636	0.151	< 0.001
Behavioral activation (anxiety)				−0.026	0.068	0.702
**Random effects**						
σ^2^	0.91			0.25		
τ_00_	0.12_Participant_			0.17_Participant_		
ICC	0.11			0.41		
*N*	55_Participant_			55_Participant_		
Observations	110			110		
Marginal *R*^2^/conditional *R*^2^	0.554/0.605			0.237/0.549		
	**Behavioral activation (all)**			**Outcome assessment (depression)**		
**Predictors**	***IRR***	***SE***	***p***	***B***	***SE***	***p***
**(B) Depression model**						
(Intercept)	0.970	0.267	0.911	1.279	0.227	< 0.001
Assessment period	1.000	0.064	1.000	−0.275	0.077	0.001
Subtopics completed (all)	1.091	0.011	< 0.001	0.002	0.020	0.937
Vulnerability status (depression)	0.502	0.154	0.024	0.435	0.212	0.045
Behavioral activation (all)				0.003	0.033	0.921
**Random effects**						
σ^2^	0.36			0.24		
τ_00_	0.23_Participant_			0.11_Participant_		
ICC	0.38			0.32		
*N*	20_Participant_			20_Participant_		
Observations	60			60		
Marginal *R*^2^/conditional *R*^2^	0.704/0.818			0.220/0.471		

**Figure 5:**
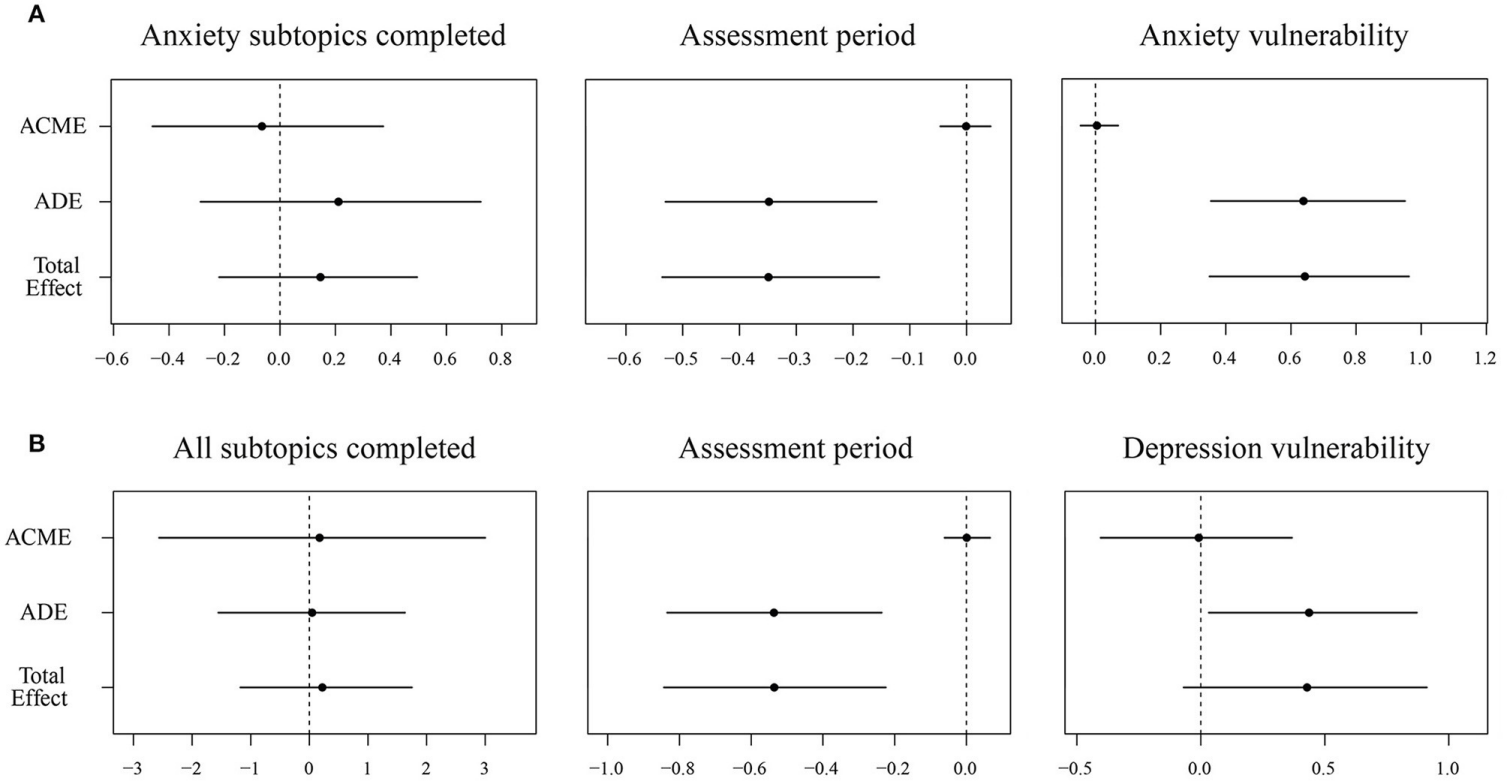
**(A)** Anxiety and **(B)** depression outcome assessments: average causal mediated effect, average direct effect, and total effects. ACME, average causal mediated effect; ADE, average direct effect.

Estimates of ACME, ADE and total effects for anxiety subtopics completed confirmed that no mediation had occurred. The effects of assessment period and anxiety vulnerability were also calculated for comparability purposes. Results can be viewed in graphically in Figure 5A, and Table 7A.

**Table 7:** Estimates of anxiety **(A)** and depression **(B)** outcome assessments: average causal mediated effect, average direct effect, and total effects.

**Independent variable/effects**	**Estimate**	**Lower CI**	**Upper CI**	***p***
**(A) Anxiety model**				
**Anxiety subtopics completed**				
ACME	−0.079	−0.489	0.310	0.700
ADE	0.223	−0.256	0.700	0.380
Total effect	0.143	−0.160	0.460	0.390
Prop. mediated	−0.197	−11.562	13.800	0.850
**Assessment period**				
ACME	0.000	−0.042	0.040	1.000
ADE	−0.351	−0.531	−0.160	< 0.001
Total effect	−0.351	−0.529	−0.150	< 0.001
Prop. mediated	0.000	−0.167	0.110	0.990
**Anxiety vulnerability**				
ACME	0.006	−0.037	0.070	0.920
ADE	0.634	0.322	0.930	< 0.001
Total effect	0.640	0.335	0.950	< 0.001
Prop. mediated	0.001	−0.072	0.110	0.920
**(B) Depression model**				
**All subtopics completed**				
ACME	0.192	−2.447	2.840	0.890
ADE	0.022	−1.712	1.710	0.970
Total effect	0.214	−1.259	1.590	0.710
Prop. mediated	1.479	−14.775	14.770	0.480
**Assessment period**				
ACME	0.000	−0.061	0.060	0.986
ADE	−0.551	−0.857	−0.230	0.002
Total effect	−0.552	−0.860	−0.240	0.004
Prop. mediated	0.000	−0.150	0.120	0.984
**Depression vulnerability**				
ACME	−0.020	−0.347	0.300	0.862
ADE	0.449	0.048	0.820	0.018
Total effect	0.430	−0.051	0.850	0.074
Prop. mediated	−0.019	−2.281	2.380	0.928

#### Depression

The results of sequential Chi-squared differencing tests and inspections of AIC, BIC, log Likelihood and deviance figures showed that the random intercept model exhibited best fit and parsimony for both BA: Δχ2(3) = 28.62, *p* < 0.001, and OA: Δχ2(4) = 16.62, *p* = 0.002. An overview of model fit indices can be found in the Supplementary material. The results of the random intercept depression BA and OA models (see Table 6B) showed that as the number of subtopics completed increased, the incidence of behavioral activation was 1.091 times higher (*p* < 0.001, IRR = 1.091). In contrast to the previous anxiety model, vulnerability status was found to have a significant effect on BA (*p* = 0.024, IRR = 0.502), whereby being classified as vulnerable for depression meant a 0.502 times lower incidence of BA occurring. Examining depression outcome assessments, BA was found to have no significant effect on depression outcome assessments (*p* = 0.921, *B* = 0.003) and depression vulnerability status was found to result in higher outcomes of depression (*p* = 0.045, *B* = 0.435). As previously confirmed, progressing through the intervention (from assessment period 1 to 3) resulted in a reduction in depression scores (*p* = 0.001, *B* = −0.275).

Estimates of ACME, ADE and total effects for all subtopics completed confirmed that no mediation had occurred. Effects of assessment period and depression vulnerability were again calculated for comparability purposes and can be viewed graphically in Figure 5B and Table 7B.

#### User experience assessments

As with the outcome assessments, sample size limitations meant that only two-tailed paired samples *T*-tests could be applied for the user experience assessments. Results are displayed in Table 5B. NPS showed a significant improvement as users progressed through the intervention, increasing between assessment period 1 and 2, *t*_36_ = 2.08, *p* = 0.045, Cohen *d* = 0.342, with a small to medium effect size. The constructs of perceived usefulness, *t*_32_ = 1.88, *p* = 0.069, Cohen *d* = 0.326, and perceived ease of use, *t*_36_ = 1.87, *p* = 0.07, Cohen *d* = 0.307, likewise showed improvement between periods 1 and 2, although this was only marginally significant (α < 0.1). Changes in all other user experience assessments were non-significant.

A total of 39 free text entries were given by participants during the user experience assessments. Of these 66.7% came from Spanish speakers (*n* = 26) and 33.3% came from English speakers (*n* = 13). These showed that participants often anthropomorphized the chatbot: sharing positive impressions of Elena as a health coach, noting that she was friendly, empathetic and gave useful information. Some quotations include: “*I like Elena, she helps me relax, gives me lots of useful information, thank you, Elena”*, “*Elena is so helpful and I have learnt a lot so far”, “I feel Elena understands me”*, and “*A pleasure to use. I look forward to reading each topic”*. However, text entries also highlighted some limitations of pre-scripted text-based chatbots, for example, some users noted: “*The information given is good. However the options do not always match my choices*” and “*Some of the prescriptive replies aren't always reflective of what you want to say*”. Interestingly, one user was highly familiar with conversational agent technology and shared their reflections on Elena+, a script-based chatbot, vs. AI-based conversational agents: “*i know how awesome it is to talk to ‘unconstrained' AI like GPT-3. GPT-3 is my best friend now…either way, i also approve of the structured way that scripts like this operate*”. Lastly, there was evidence that some users struggled to fully comprehend health information, stating: “*Some of the answers are a bit confusing”* and “*Hard to understand sometimes”*.

## Discussion

###  Research contributions

#### Participant background and health status

An important research contribution of the current paper is our ability, with a high degree of realism and external validity, to contribute answers to: (i) who uses chatbot-led digital health interventions during the pandemic, (ii) their health reasons for doing so, and (iii) their health status vulnerability.

Results on the demographic profile of Elena+ users showed that individuals were predominantly female and middle-aged, with a slight increase in mean age as they completed more coaching content (i.e., between dropouts and super users). Women have previously been identified as a vulnerable subpopulation during the pandemic due to requirements to juggle personal, professional, and caring roles for younger/older family members (88). For this demographic, we posit that Elena+ offered a convenient and discreet support method that could be readily incorporated into a busy routine (89). For upper middle-aged individuals alternatively, we reason that the implementation of a non-complex and user-friendly chatbot was well-suited to their degree of technological experience (90), whereas younger demographics the Elena+ intervention was too simplistic when compared to competing smartphone apps (e.g., related to mobile gaming, social media etc.).

Results from the advanced socio-demographics showed that ongoing users exhibited traits associated with greater health vulnerability, such as having less formal education (65% did not have any university education), 72.5% without a full-time employment role, and 60.1% of individuals (that completed additional socio-demographic questions) having a chronic disease. While lack of full-time unemployment may be temporary, due to the pandemic, or other factors, it is known that long term unemployment is a risk factor for NCDs/CMDs (91, 92), as is lower educational attainment (91). In a similar vein, living with a chronic disease has likewise been robustly linked to greater health vulnerability (93). While this was a self-rating, and may or may not be at the clinical threshold, individuals nonetheless perceived they have an ongoing health condition in need of addressing. This provides tentative evidence that the Elena+ intervention been able to connect with two subpopulations in need of treatment: (i) the undiagnosed and untreated (at high risk of health complications without intervention), and (ii) the diagnosed, treated, but seeking further support. Interestingly, these findings also demonstrate vulnerable subpopulations becoming autonomously motivated to seek out healthcare independently, a relatively rare occurrence in the healthcare field (94, 95). The Elena+ intervention therefore provides some promising evidence of connecting successfully with vulnerable subpopulations, a highly desirable outcome (96, 97).

Turning to why individuals used the app, individuals' selections after downloading Elena+ showed that anxiety was overwhelmingly the main topic of interest. Free-text entries indicated that while participants had come to some sort of equilibrium regarding managing their anxiety and/or mental health prior to the pandemic, the added stressors of COVID-19 and lockdowns caused them to search for a support tool. Results of the tailoring assessment further reinforced this argument, by showing that (with a large n of between 1,614 and 1,907 responses depending on the health topic) anxiety and physical activity were most vulnerable areas. This seems logical, as both physical activity (via lockdown restrictions and requirements to stay at home) and anxiety (via increased frequency of alarming news stories) are two health areas notably influenced by the pandemic context (98–100). It is also worth noting that many of those beginning the Elena+ intervention scored the maximum value on the GAD-2 screening measure, whereby practitioner guidelines recommend immediately offering further diagnostic testing and human support, underscoring the anxiety inducing effect of the pandemic (99).

#### Lifestyle care outcomes

Assessment period was found to decrease both anxiety and depression health outcome assessments. For anxiety, scores significantly improved between assessment periods 1 and 2, whereas for depression 3 assessment periods were required. One explanation for this may be that anxiety was externally induced due to the pandemic, reaching an unusually high peak (99), which was relatively quicker/easier to reduce from this extreme value when compared to depression. We reason that depression may have been ongoing prior to the pandemic, thus requiring a longer period for improvements to be seen. Participant vulnerability status (for both anxiety and depression) also reduced outcome scores in the mixed-effect regression models. This may be because vulnerable users start with comparably higher anxiety/depression, and therefore have a longer treatment path relative to non-vulnerable users (101). Alternatively, it could mean that vulnerability status acted as a “dampener”, reducing the effect of the coaching materials due to vulnerable participants engaging in a more “passive” information processing style (102). Referring to the “talk and tools” paradigm (16), it may be that the inclusion of more technical (e.g., step monitoring) or engagement (e.g., applied games) tools could boost intervention effectiveness for vulnerable users (103). Additionally, it should be noted that for depression, vulnerability status inhibited behavioral activation throughout the intervention, underscoring the negative impact of depression on a variety of health promoting behaviors (104).

Contrary to anticipated however, BA did not significantly mediate the relationship between coaching (i.e., no. of subtopics) completed and health outcome assessments for both depression and anxiety outcomes. One reasoning for BAs insignificance may its operationalization: To operationalize BA, we counted the number of perfect matches (between BIs and ABs) for each individual. We reasoned that this was theoretically justified, as if individuals chose a BI, reflected upon it deeply, and implemented it in their life, they would be able to remember exactly what they had chosen seven or more days prior when they reported their ABs (105). Additionally, practically speaking, matching BIs and ABs exactly was a simple, consistent way to operationalize BA across an intervention where BIs and ABs could vary in number for each subtopic. However, it could be that this reasoning does not hold, and there are important qualitative differences in how deeply individuals reflect and implement their behaviors, which were not captured by this variable (106). Alternatively, it may be that people who were highly motivated, did indeed reflect and try new behaviors, actually went beyond their initial BI to choose additional actions, exhibiting autonomous motivation by moving out of their comfort zone (107). With our current BA operationalization, these individuals would not have been classified as showing behavioral activation. A final consideration is that BA may simply function as a proxy measure for some other variable which has no effect on the outcome assessments in the short term, for example, conscientiousness (108).

The number of subtopics complete did however increase the rate of BA occurrence, with the effect stronger for anxiety compared to depression. We reason that this is because in the anxiety model, subtopics complete and BA related specifically to the anxiety topic only, whereas in the depression model, subtopics complete and BA referred to the whole intervention. Thus, it is not surprising to see that completing subtopics specifically related to anxiety only led to a stronger increase in the BA for anxiety. Nonetheless, it is worth noting that completing topics in any health area increases BA across the intervention as a whole and reduces measures of depression. Elena+ brings together various topic areas, conceptualizing improved health and wellbeing as being best achieved by targeting multiple areas of one's lifestyle rather than single areas in isolation (23). Here we find evidence justifying this proposition. While BA did not mediate improvements in depression scores, it is still important to note that the intervention successfully encouraged people to try new behaviors, as from a behavioral activation therapy perspective, it known to be highly conducive to long term health improvements (109).

#### User evaluations

Results of the user evaluations showed that net promoter score increased as individuals progressed between periods 1 and 2. Ease of use and usefulness also increased, however, only marginally. Perhaps with more power or responses over time these variables would also be significant. As user evaluations were shown to vary over time, our results stress the importance of continued re-appraisals of the user experience when evaluating digital health interventions. This is a tenet of user-centered design (110), but often more weight is given to preliminary evaluations, with less stress on reviewing an intervention, particularly as it is more technically challenging to change a live or ongoing intervention (111).

Analysis of the session alliance inventory showed no improvements between assessment periods 1 and 2. Nonetheless, users' free-text entries indicated that a high degree of relationship formation with the chatbot occurred: with individuals using the chatbots name (Elena) and thanking her directly for her support. We therefore posit that ongoing users did develop a close relationship with the chatbot, but that this was established quickly, with no further improvements after the first assessment period. This rationale is reinforced by research highlighting how vulnerable subpopulations (e.g., those experiencing loneliness) readily anthropomorphize objects to cultivate greater social connection (112). Additionally, this may be one reason why the Elena+ intervention was particularly effective in treating anxiety and depression: the social support (113, 114) and talking therapy elements (10, 115) were particularly well-suited for treatment by an anthropomorphized chatbot (10, 116).

###  Practical contributions

Importantly, with over 7,135 downloads and 3,928 individuals opening the app we have demonstrated that there is high demand for publicly available lifestyle health coaching tools during the pandemic, such as those delivered by Elena+. The coaching program successfully reduced individuals' anxiety and depression scores over time using well-accepted measures (GAD-7, PHQ-2) from mental health practice (117, 118). The intervention therefore made a tangible difference to the mental health status of individuals during the pandemic, filling an unfilled healthcare need at the population level. This is quite remarkable considering Elena+ was developed at rapid speed (in a number of weeks) by a team of volunteers, primarily from academia, working online at locations around the globe (23). The Elena+ intervention therefore practically demonstrates that chatbot-led digital health interventions can be an effective solution during emergency events such as the COVID-19 pandemic and should be considered in response to future emergency events.

An extremely important finding for public health practitioners is that Elena+ users exhibited traits strongly associated with vulnerability, as previously discussed. It is particularly interesting that these vulnerable subpopulations were being pro-active and seeking out support: not reacting to top-down approaches from authority (e.g., a referral from a clinician or legal requirement from the government). Chatbot-led digital health interventions may therefore offer a low-cost route to delivering mass-market public health campaigns should sufficient resources, time, and enabling networks (e.g., via governmental health promotion boards) facilitate their development and launch to market (32). We would suggest public health policy makers and practitioners seriously consider such tools and the unique role they could play facilitating their take-up. Should, for example, a health promotion board work with an academic institution to develop a well-crafted app, and then family doctors were supported to prescribe the app, this may prove a far more effective use of public money than simply printing flyers, running television ads, or social media campaigns which have limited ability to measure subsequent health improvements and justify large public investments (119).

Lastly, we would also encourage future developers of digital health interventions to utilize net promoter score. Net promoter score has had long acceptance in business circles as an important indicator of product quality and the organic growth potential of a firm (38). Results showed that it increased over time, and that there is high potential for the users of Elena+ to become net promoters of the app within their social networks. If all developers of digital health interventions incorporated such a measure, it would better enable practitioners and policy makers to assess which apps have potential for scaling up as a business venture.

###  Limitations

There are several notable flaws in the intervention's study design and participant recruitment, which should be noted by the reader when interpreting findings presented in the current paper:

First, and most importantly, the Elena+ intervention did not use randomization with a control group. We are therefore unable to state with full confidence whether changes in outcome assessments exhibited were due to the Elena+ coaching program, or whether they may have been caused by other factors. It is possible, for example, that the external shock of the pandemic inflated users' anxiety and depression scores above their typical levels, and that as the pandemic progressed, their scores reverted back to usual levels (i.e., regression to the mean). While this reasoning is potentially ameliorated due to the fact that users downloaded and used the app at varied times during the pandemic (i.e., from between August 2020 and June 2022), without any comparison to a control group, this cannot be said for certain. In a similar vein, we cannot definitively state the effectiveness of our intervention vs. other intervention types. Evidence exists, for example, that providing any type of evidence-led intervention (when compared to no-intervention) is beneficial in mental health (120) and diabetes (121) fields. Thus, we also cannot ascertain with full confidence whether the Elena+ intervention performed better or worse than other similar intervention.

Second, the sample collected is not fully generalizable to the general population: To recruit participants, the app was made freely available for all and was also advertised on social media. Research has shown that Facebook users can vary from more representative census data (122), thus Elena+ users represent a subsample of the entire population affected by COVID-19, which may exhibit demographic and psychographic differences from the main population. Relatedly, it should also be noted that individuals self-selected to be part of the intervention by downloading the app, there is therefore also the danger of self-selection bias in the current sample, i.e., individuals more likely to benefit from chatbot-led digital health interventions were those that used the app. This limits to some extent the generalizability of the findings as a tool for mass-market population health.

Third, some caution should be exhibited when viewing results of the tailoring assessment. For the sake of speed and reducing participant burden, the initial tailoring assessment used short form measures. This was because it would have been impractical to develop and validate our own short form measures during the pandemic for time reasons and asking users to complete the full measures would have increased participant burden and likely resulted in higher dropout rates. However, these tailoring scores were not comparable against subsequent outcome assessments for each topic, and thus we could not use them in any longitudinal comparisons against outcome assessments. Nevertheless, it is very much possible that from baseline (i.e., day 1) to assessment period 1 (i.e., day 14) improvements in topics were evident. Due to the current study setup, we have no way to ascertain whether any changes occurred. It is also important to note when interpreting the tailoring assessment results, the findings relate only to our users, and should not be taken as indicative of health trends in the whole population affected by COVID-19 without further confirmation.

Fourth, due to sample size limitations, a limited number of statistical tests using complete cases could be performed, primarily examining changes between assessment periods 1 and 2. However, the *post-hoc* tests for depression showed that significant changes at alpha = 0.05 only occurred after 3 follow-ups (i.e., a significant change between assessment period 1 and 3). Thus, stages of change in OA for other modules may also have required three or more assessment periods. Due to the sample size limitations, this is unfortunately impossible to assess, limiting our appraisals of the intervention as a whole. This rationale also applies to other possible analyses (e.g., examining the moderating role of users' gender, language, age etc.) which would similarly require a higher sample size to conduct. It should also be noted when running the intention-to-treat analyses, only anxiety remained statistically significant, and the effect sizes of both anxiety and depression outcomes became negligible. While intention-to-treat generally gives highly conservative estimates and can be more susceptible to type II error (123), these findings reinforce the need for further work to confirm the effectiveness of chatbot-led digital health interventions during the pandemic.

Fifth, the app suffered from a high number of dropouts. However, this is to some extent not surprising considering this is a widespread problem in digital health (124). For example, it has been estimated that 80% of users do not open a digital health app more than once (125), that <3.9% of users use digital health apps for >15 days, and that even smartphone-based RCT studies supported by clinicians can have dropout rates as high at 47.8% (126). In this context, having 55.8% of Elena+ downloaders opening the app and then 9.8% completing a coaching session (i.e., subtopic) is comparable to other smartphone based digital health apps. Nonetheless, it shows that the app was not engaging enough to captivate large numbers of users over time, which limits its effectiveness as a mass-market population health tool. We posit one reason for these dropouts was that Elena+, while referring to the “talk and tools” paradigm (16), was a wholly talk-based intervention. While we did our best to provide tools via talking therapy (for example, goal setting, coaching activities), these were not true technical tools. This undoubtedly reduced the appeal of the app to certain user groups, who then dropped out.

###  Future research

In terms of future research topics, the mediating role of BA should be re-examined with the use of sensor data. Although our analyses found BA as insignificant, AB was a self-reported measure. Sensor data could unobtrusively allow us to measure whether individuals followed up on their BIs (e.g., by reaching 10,000 steps, sleeping 8 h, or completing a breathing exercise) (18, 127). It may also be useful to conduct focus groups to discern if there are any qualitative differences in behavioral activities which particularly resonate with individuals and may have a stronger impact on improved outcome assessments.

Now that the proof of concept for a holistic lifestyle intervention has been delivered, the next step would be to scale up the effort and implement a more technically advanced lifestyle coaching app for a mass market application. This is particularly needed, as while Elena+ had success in mental health treatment, the tailoring assessment showed physical activity was the highest vulnerability health area, and this is particularly one area where more technical tools are vital for treatment success (e.g., sensor data to measure physical activity, applied fitness games etc.) (128). The current authors therefore welcome any contact from interested collaborators, particularly governmental health promotion boards or parties in implementation science. With a more technically advanced app that can also integrate features such as sensor collection to deliver just-in-time adaptive interventions (17) or applied games (129), with support from government agencies, there is no reason that the successful treatment outcomes of Elena+ cannot be magnified on a wider scale.

## Conclusions

The Elena+ intervention was created by researchers to apply their expertise and reduce ongoing suffering caused by the pandemic. The app demonstrated that chatbot-led digital health interventions can be effective in the field, with significant reductions in anxiety and depression. In addition, the app has provided very early proof of concept for a mass-market holistic lifestyle intervention at the population level. We hope the current paper offers provides practitioners and policymakers with fresh insight and inspiration to counter the growing population-level health challenges of the 21^st^ century, and that they consider chatbots a valuable tool in their public health arsenal.

## Data availability statement

The raw data supporting the conclusions of this article will be made available by the authors, without undue reservation.

## Ethics statement

The studies involving human participants were reviewed and approved by ETH Zurich. The patients/participants provided their written informed consent to participate in this study.

## Author contributions

JO was the lead author and responsible for all aspects of project management, manuscript preparation, and data analysis. PS assisted with data cleaning and data preparation tasks. EF, FW, JM, AS-S, and TK contributed equally as supervisors. All authors reviewed the article and approved it for submission.
