# Supplementary material for: Can digital health researchers make a difference during the pandemic? Results of the single-arm, chatbot-led Elena+: Care for COVID-19 interventional study
Source: Front Public Health. 2023 Aug 25;11:1185702. doi: 10.3389/fpubh.2023.1185702 (PMC10485275; doi:10.3389/fpubh.2023.1185702)
Supplement: Supplementary file 1 [file Data_Sheet_1.PDF]

frontiers

Supplementary Material

1 Elena+ coaching topics and subtopics.

Topic	Beginner Subtopics	Intermediate+ Subtopics
COVID-19	What is COVID-19 and what are coronaviruses?	What are pandemics and why do they occur?
	What are the symptoms and how do they differ from the flu?	How and when should I self-isolate?
	How is COVID-19 coronavirus spread?	How can I get tested/diagnosed for COVID-19?
	What groups are most at risk?	Are hospitals/medical facilities safe to visit?
	How can we prevent the spread?	More advanced information on preventing transmission/catching COVID-19
Physical Activity	What is physical activity and how much should I do?	How does physical activity affect my immune system?
	What are the benefits of being active?	Safety, inspiration, and fitness goals during COVID-19
	Getting more active during COVID-19	How can I improve my fitness?
	Safe exercising during COVID-19	How can I maximize the benefits of physical activity?
Sleep	Why is sleep important?	What is sleep hygiene?
	How does healthy sleep help to protect me from COVID-19?	What hinders and helps good sleep?
	Is good sleep important for my mental health?	How does poor sleep put me at risk for COVID-19?
	What happens if I do not sleep well?	How can I manage to sleep well during confinement?
	Can anxiety, stress, and poor sleep cause COVID-19?	
Diet & Nutrition	Unhealthy food hazards	
	The positive effects of a nutrition-rich diet	
	Preparing meals with the daily dozen	

Topic	Subtopics
Anxiety	What is anxiety and why is it hard to control?
	COVID-19, risk perception and anxiety
	How can I control my anxiety?

	Breathing away anxiety
	Confinement and anxiety
Loneliness	What is loneliness?
	Can loneliness make you sick?
	How can we deal with loneliness?
Mental resources	The fundamentals of mental resources
	The functions of mental resources
	The neuroscience behind mental resources
	Identifying our mental resources
	Activating our mental resources

2 Timing schedule of outcome assessments and actual behaviors in days.

**Topic**	**Actual Behavior Follow-Ups:**				**Assessments:**					**-/+ Days**
	1st	2nd	3rd	4th	1st	2nd	3rd	4th	5th	
Anxiety	7	21	35	77	14	28	42	84	126	0
Mental resources	8	22	36	78	15	29	43	85	127	+1
Loneliness	9	23	37	79	16	30	44	86	128	+2
Sleep	10	24	38	80	17	31	45	87	129	+3
Physical activity	6	20	34	76	13	27	41	83	125	-1
Diet & nutrition	5	19	33	75	12	26	40	82	124	-2
COVID-19 information	4	18	32	75	11	25	39	81	123	-3

3 Example screenshot of behavioral intention (BI) setting.

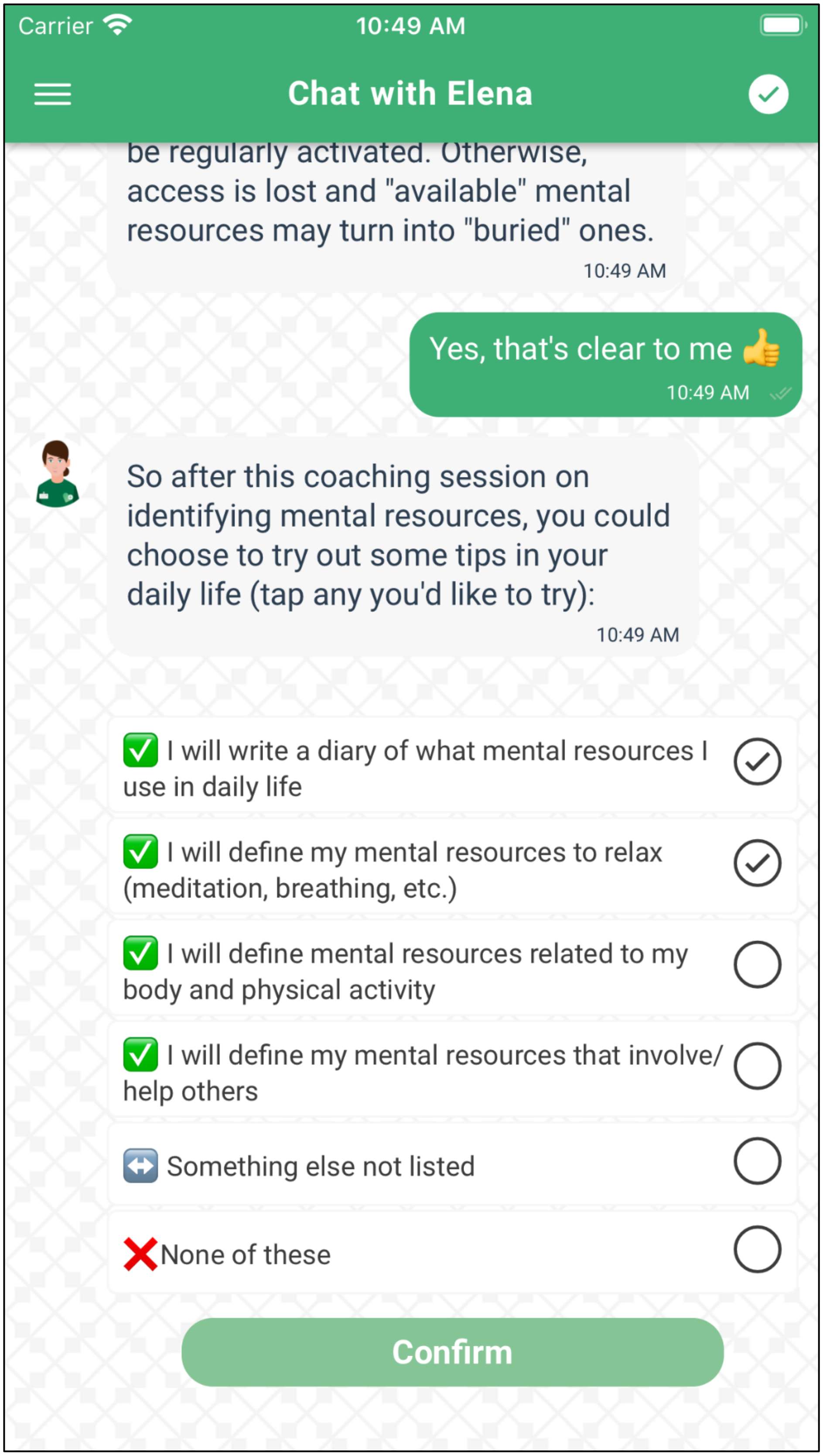

4 Marker variables

To detail the users' background and their usage experience, we collected "marker variables" (i.e., user choices in dialogue with the chatbot) throughout the intervention. Marker variables described the user profile, usage experience and health background of users in a fully objective fashion, as they are collected silently in the background as users progress through the dialogues. Examples include a humor marker variable, that collects whether a user asked the chatbot for a joke or not, or literacy marker variable, that indicates whether a user understood an explanation of not. Marker variables were operationalized by having the second user dialogue choice indicative of a certain state. For example, during a sleep subtopic, the chatbot states: "According to the Sleep Foundation, without sufficient sleep 💤, your body makes fewer cytokines, a type of protein that is important for your immune response by targeting infection and inflammation." User answer options were: "Good to know" and "Hmm that's a bit confusing 🤔". If a user selected the second option for this literacy marker variable, they would be classified as demonstrating lower health literacy. Table 1 contains the list of marker variables extracted for analysis and their definitions.

Table 1. Marker variables

Marker variable	Description
Anxiety marker	Indicative of being in an anxious state or often experiencing anxious feelings
Loneliness marker	Indicative or being lonely or often feeling lonely
Sleep marker	Indicative of having had poor sleep recently or often experiencing poor sleep
Depression marker	Indicative of currently having a low mood or often feeling low
Literacy marker	Indicative of confusion related to health explanations and/or poor health literacy
Humor marker	Finding conversational turns humorous or asking the chatbot for a joke
More coaching marker	Indicative of wanting to do more coaching in new health areas
Sedentary marker	Indicative of engaging in sedentary behavior

We used marker variables to descriptively outline users and their usage experiences, classified into four user types: (i) dropouts (i.e., opening the app and beginning a dialogue but 0 completed subtopics), (ii) tentative users (completing one or more subtopic), (iii) users (completing five or more subtopics), or (iv) super-users (completing 30 or more subtopics). For each marker variable, we calculated the "selection rate" in percentage terms. This is the number of times an individual selected the second answer option for a marker variable type, divided by the total number of times an individual came across this marker variable type during the intervention. For example, the number of times an individual was classified as showing anxiety (i.e., by selecting the second answer option for anxiety markers), divided by the total number of times an individual saw anxiety markers (selecting any answer option) in the whole intervention. Selection rate is divided into four classifications: (i) never (selected the marker variable 0% of the times possible), (ii) less than half the time (selected the marker variable 1-49% of the times possible), (iii) more than half the time (selected the marker variable 50-99% of the times possible) and (iv) always (selected the marker variable 100% of the times possible).

Results of the marker variable classification are displayed in Table 2. As dropouts (i.e., individuals that did not complete any subtopics) had very few chances to select any marker variables, they were excluded from the table. Examining users and super users (i.e., users that completed 5-43 subtopics, and thus had given responses to marker variables multiple times) showed some indications of consistent vulnerability amongst Elena+ users. For example, 23.4% of users and 22.2% of super users always selected answers indicating they experienced loneliness, 12.3% of users and 33.3% of super users always selected answers indicating they experienced low mood or depression, and 11.2% of users and 22.2% of super users always selected answers indicating they have trouble sleeping. In summary, the vulnerability of users using the Elena+ app was evidenced, with marker variables showing a notable percentage of users and super users always selecting answer options indicating loneliness, depression, and poor sleep quality.

Table 2. Marker variable selection rate amongst different user groups

**Marker Variables**	**Selection rate**	**Tentative users**	**Users**	**Super users**
**Subtopics complete (n)**		1-4	5-29	30-43
**Participants (n, %)**		590 (8.3)	98 (1.4)	9 (0.1)
**Anxiety marker (n, %)**	Never	489 (83)	36 (36.7)	2 (25)
	Less than half the time	52 (8.8)	21 (21.4)	5 (50)
	More than half the time	1 (0.01)	13 (13.2)	2 (25)
	Always	48 (8.1)	28 (28.5)	0 (0)
**Literacy marker (n, %)**	Never	323 (54.7)	6 (6.1)	0 (0)
	Less than half the time	159 (27)	70 (71.4)	7 (77.8)
	More than half the time	37 (6.3)	20 (20.4)	2 (22.2)
	Always	71 (12)	2 (2)	0 (0)
**Humor marker (n, %)**	Never	428 (72.5)	26 (26.5)	0 (0)
	Less than half the time	82 (13.9)	63 (64.2)	8 (88.9)
	More than half the time	21 (3.5)	9 (9.1)	1 (11.1)
	Always	59 (10)	0 (0)	0 (0)
**More Coaching marker (n, %)**	Never	465 (78.8)	27 (27.5)	2 (22.2)
	Less than half the time	38 (6.4)	41 (41.8)	3 (33.3)
	More than half the time	7 (7.1)	17 (17.3)	4 (44.4)
	Always	80 (13.5)	13 (13.2)	0 (0)
**Loneliness marker (n, %)**	Never	551 (93.3)	69 (70.4)	5 (55.5)
	Less than half the time	1 (0.16)	6 (6.1)	2 (22.2)
	More than half the time	0 (0)	0 (0)	0 (0)
	Always	38 (6.4)	23 (23.4)	2 (22.2)
**Sleep marker (n, %)**	Never	584 (99)	86 (87.7)	3 (33.3)
	Less than half the time	0 (0)	1 (1)	4 (44.4)
	More than half the time	0 (0)	0 (0)	0 (0)
	Always	6 (1)	11 (11.2)	2 (22.2)
**Depression marker (n, %)**	Never	580 (98.3)	85 (86.6)	6 (66.6)
	Less than half the time	0 (0)	1 (1)	0 (0)
	More than half the time	0 (0)	0 (0)	0 (0)
	Always	10 (1.7)	12 (12.3)	3 (33.3)
**Sedentary marker**	Never	560 (95)	65 (66.3)	0 (0)

**(n, %)**	Less than half the time	6 (1)	15 (15.3)	6 (66.6)
	More than half the time	5 (0.8)	2 (2)	3 (33.3)
	Always	19 (3.2)	16 (16.3)	0 (0)

While marker variables were not a core part of the study design during the development of Elena+, they nonetheless provided some interesting descriptive insight. We would therefore encourage other researchers to consider implementing marker variables in the future. One practical research example could be to divide marker variables into "pull" (i.e., engagement) and "push" (i.e., vulnerability-based) types. Pull marker variables could describe entertainment or engagement factors that "pull" users into an immersing coaching experience, for example, engaging in humorous turns with the chatbot or stating that an activity was fun. Push marker variables could indicate health factors that "push" individuals into using an intervention: for example, having poor coping behaviors (e.g., smoking, drinking alcohol) or exhibiting lower health literacy (e.g., not understanding information). If gathered in a balanced manner, at sufficient scale, and with sufficient sample size, such markers may give fresh insight into usage of chatbot-led digital health interventions.

5 Frequency counts for categories with example quotes.

Table 1. Category classification: reason for downloading Elena+

**English-speaking participants**		**Spanish-speaking participants**	
**Words**	**N (%)**	**Words**	**N (%)**
Mental health	81 (29.6)	Mental health	205 (38.0)
Anxiety	71 (25.9)	COVID care	115 (21.3)
Loneliness	28 (10.2)	Anxiety	80 (14.8)
COVID care	17 (6.2)	Diet	74 (13.7)
Diet	16 (5.8)	Diet and PA	17 (3.1)
Diet and PA	14 (5.1)	Loneliness	11 (2.0)
Contracted COVID	12 (4.4)	Sleep	8 (1.5)
No reason	10 (3.6)	Diet and Anxiety	7 (1.3)
Support	8 (2.9)	PA	4 (0.7)
General knowledge	5 (1.8)	General knowledge	3 (0.6)
PA	3 (1.1)	Contracted COVID	3 (0.6)
Chronic disease	3 (1.1)	Mental health and sleep	3 (0.6)
Helping others	3 (1.1)	Health	2 (0.4)
Sleep	1 (0.4)	Helping others	2 (0.4)
Curiosity	1 (0.4)	No reason	2 (0.4)
Mental health, and PA	1 (0.4)	To receive help	2 (0.4)
		COVID care and diet	1 (0.2)
		Mental health and diet	1 (0.2)
**Total**	**274 (100)**		**540 (100)**

Note. *PA = physical activity*

Table 2. Categories and example quotes: Reasons for downloading Elena+

**English-speaking participants**		**Spanish-speaking participants**	
**Category**	**Quotations**		**Quotations**
Mental health	"I don't feel like talking to people" [3C]; "I want to be happy and productive. At the moment i am often procrastinating" [17Q];" "I suffer from depression anxiety and stress" [90L]";" Stress with covid 19; "Staying resilient, recognising that there are difficult days and the future is unclear and remember to practice self-care"	Mental health	"I feel anxious, I fell into depression again, I am insecure, I am afraid, stressed, insomnia, tired, emotionally empty and I want to die'[17Qs];'To be sure that quarantine does not affect emotional health" [52Zs];" Keep calm in these moments" [70Rs]; "I think I suffer from depression"[153Ys].
Anxiety	"I suffer from anxiety for some years now. Even though during the pandemic outbreak my anxiety was pretty well under control, i feel now how i struggle at nights with very intense fears" [8H]; "I have a lot of anxiety"; "Im anxious all the time, and I panic" [30D]; "I need help with anxiety "	COVID care	"How to handle COVID-19" [49WS];" knowing how to control it" [62Js]; "to know how can I fight it"[84Fs];"to know how to take care of myself"[126Vs];
Loneliness	"I feel lonely" [31F]; "I live alone" [40N]; "Feeling isolated in lockdown" [59G];" Im alone"; "social isolation"	Anxiety	"I have anxiety" [77Ys];" I suffer from anxiety'[85Gs]; "Calming my anxiety"[74Vs]; "I feel anxious all the time "[89Ks]; "how to control my anxiety [141Ks]
COVID care	"How to manage it and make it go away"; "i don´t want to suffer or die of covid-19"	Diet	"I am coming out of this virus and I need to know about a diet" [127Ws]; "Healthy food for my family, we are fat, with diabetes and hypertension" [143Ms]; "nutrition" [284Ts]; "Diet to increase my defenses" [477Ns].
Diet	"I want to eat healthy" [4D]; "Good nutrition" [85G];" I want to eat healthier."	Diet and PA	"Lose weight" [57Es]; "What to eat to not gain weight, and exercise" [54Bs]; "I am very fat" [53Xs]; "Obesity"[199Ss]
Diet and PA	"Need to lose weight" [48V]; "Lose weight" [20T]; "Diet & exercise"	Loneliness	"Being lockdown and getting along" [116Ls]; "social isolation"; "I feel very alone"; "I feel alone many times"
Contracted COVID	"Because I suffer from it" [50X]; "I have been suffering from it" [19S]; "I have it'[104Z]	Sleep	"Insomnia" [323Fs]; "I can't sleep"[412Ds]; "Because, I can't I sleep at night"[504Hs]; "After suffering from covid I can't fall asleep"[530Es]
No reason	"I don't know" [10J]; "Not sure" [56D]	Diet and anxiety	"During this situation my anxiety is to eat more, and so I am gaining weight, this affect my blood pressure"[315Gs]
Support	"The possibility to make use of resources i already have" [42P]; "Need help to deal with it"; "help" [92N]	PA	"Exercise"[219Ws]; "I want to exercise"[454Qs]
General knowledge	"Learning" [43Q]; "Just sounded the most interesting"[225R];	General knowledge	"Well, to learn more about the subject" [134Ds]
PA	"I like sports" [2B]; "Gain muscles" [44R]	Got COVID	"Because I suffer from it" [66Ns]
Chronic disease	"Coping with chronic pain with operation waiting list postponed"; "Previous diagnosis"[213W]	Mental health and sleep	"I feel very anxious and sometimes I have trouble sleeping." [106Bs]
Helping others	"I'm a health care Assistant" [12L]; "Its not about me – it's my daughter"	Health	"health"[557BS]; "because health"
Sleep	"Poor sleep"[211A]	Helping others	"Knowing how to help myself and help others"[502As]
Curiosity	"Just curious"[251F]	No reason	"I do not know, I like it"[368Fs];" "I don't know" " [6Fs]
Mental health and PA	"Interested to have some useful techniques to use for myself. Also interested in physical care too and stress and working from home how to stay sane"[204G]	To receive help	"Receive help" [506Gs]
		COVID care and diet	"If I stay healthy it is easier to resist covid and also because in these days of pandemic I have gained weight" [400Rs]
		Mental health and diet	"Mental health, nutrition, anxiety and grief management" [114Js]

6 Frequency counts of chronic disease category classification.

Table 1. Chronic disease: category classification

English-speaking participants		Spanish- speaking participants	
**Words**	**n (%)**	**Words**	**n (%)**
Comorbidity	15 (34.1)	Comorbidity	21 (35.0)
Anxiety	12 (27.3)	Anxiety	13 (21.7)
Depression	4 (9.1)	Diabetes	10 (16.7)
Asthma	2 (4.5)	Depression	7 (11.7)
Diabetes	2 (2.2)	Hypertension	3 (5.0)
Autism	1 (2.3)	Asthma	2 (3.3)
Cancer	1 (2.3)	Dyslipidemia	1 (1.7)
Chronic disease	1 (2.3)	Hypothyroidism	1 (1.7)
Fibromyalgia	1 (2.3)	Migraine	1 (1.7)
Heart condition	1 (2.3)	Osteoarthritis	1 (1.7)
Meniere’s disease	1 (2.3)		
Osteo pain	1 (2.3)		
P.T.S. D	1 (2.3)		
Rheumatoid arthritis	1 (2.3)		
**Total**	**44 (100%)**		**60(100%)**

7 Frequency of tailoring assessment scores

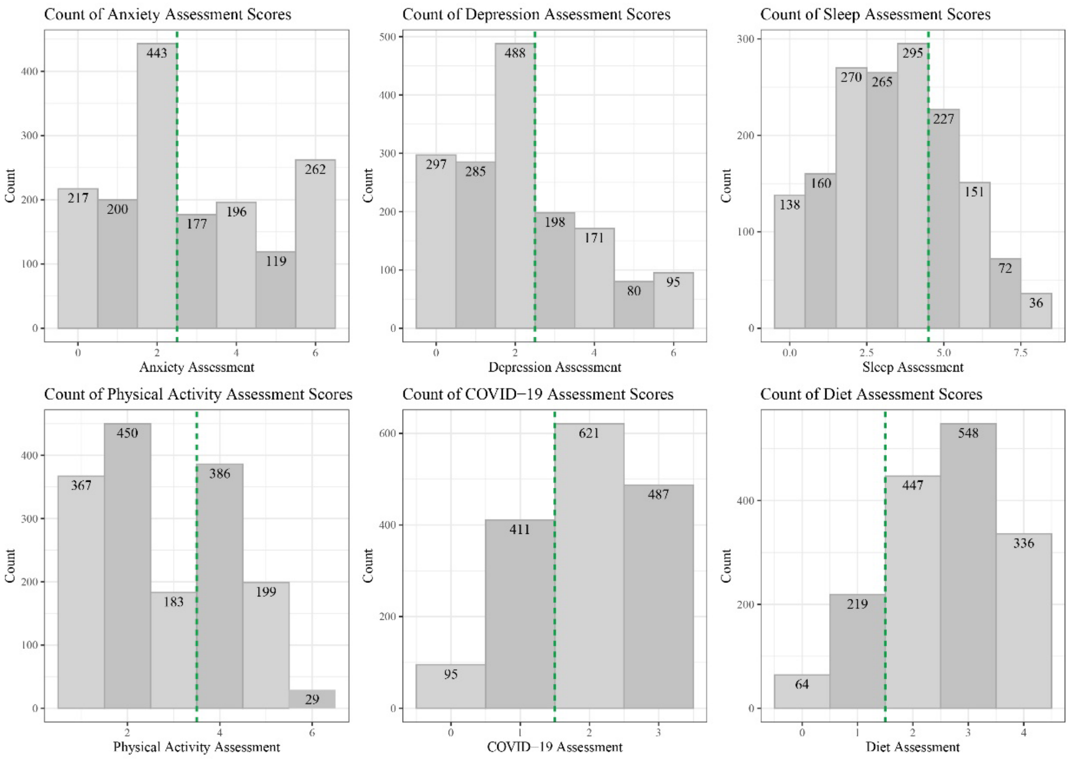

8 Anxiety mixed effects model fit indices

**Dependent variable**	Specification	AIC	BIC	log Likelihood	Deviance	χ2 difference	df	p
Anxiety behavioral activation	Intercept only	305.91	311.31	-150.96	301.91			
	Random intercept	259.47	272.97	-124.73	249.47	52.44	3	.<001
	Random slope	260.24	273.74	-125.12	250.24	0	0	-
	Random intercept and slope	259.50	278.40	-122.75	245.50	4.74	2	.09
Anxiety outcome assessments	Intercept only	236.12	244.22	-115.06	230.12			
	Random intercept	214.87	233.78	-100.44	200.87	29.25	4	.<001
	Random slope	220.44	239.35	-103.22	206.44	0	0	-
	Random intercept and slope	-	-	-	-	-	-	-

9 Depression mixed effects model fit indices

**Dependent variable**	Specification	AIC	BIC	log Likelihood	Deviance	χ2 difference	df	p
Depression behavioral activation	Intercept only	248.31	252.50	-122.15	244.31			
	Random intercept	225.69	236.16	-107.84	215.69	28.62	3	<.001
	Random slope	236.54	247.01	-113.27	226.54	0	0	-
	Random intercept and slope	232.42	253.36	-106.21	212.42	14.12	5	.014
Depression outcome assessments	Intercept only	119.12	125.41	-56.562	113.125			
	Random intercept	110.50	125.17	-48.252	96.505	16.62	4	.002
	Random slope	113.59	128.25	-49.794	99.588	0	0	-
	Random intercept and slope	114.06	132.91	-48.032	96.064	3.52	2	.172
